# Local administration of curcumin-loaded nanoparticles effectively inhibits inflammation and bone resorption associated with experimental periodontal disease

**DOI:** 10.1038/s41598-018-24866-2

**Published:** 2018-04-27

**Authors:** Laura M. G. Zambrano, Dayane A. Brandao, Fernanda R. G. Rocha, Raquel P. Marsiglio, Ieda B. Longo, Fernando L. Primo, Antonio C. Tedesco, Morgana R. Guimaraes-Stabili, Carlos Rossa Junior

**Affiliations:** 1 Department of Diagnosis and Surgery, School of Dentistry at Araraquara-Univ Estadual Paulista (UNESP), Araraquara, SP Brazil; 2 Faculty of Pharmaceutical Sciences of Araraquara, Department of Bioprocess and Biotechnology, Sao Paulo State University (UNESP), Araraquara, SP Brazil; 3 Department of Chemistry, Center of Nanotechnology and Tissue Engineering-Photobiology and Photomedicine Research Group, Faculty of Philosophy, Sciences and Letters of Ribeirao Preto, University of Sao Paulo, Ribeirao Preto, SP Brazil

## Abstract

There is evidence indicating that curcumin has multiple biological activities, including anti-inflammatory properties. *In vitro* and *in vivo* studies demonstrate that curcumin may attenuate inflammation and the connective tissue destruction associated with periodontal disease. Most of these studies use systemic administration, and considering the site-specific nature of periodontal disease and also the poor pharmacodynamic properties of curcumin, we conducted this proof of principle study to assess the biological effect of the local administration of curcumin in a nanoparticle vehicle on experimental periodontal disease. We used 16 rats divided into two groups of 8 animals according to the induction of experimental periodontal disease by bilateral injections of LPS or of the vehicle control directly into the gingival tissues 3×/week for 4 weeks. The same volume of curcumin-loaded nanoparticles or of nanoparticle vehicle was injected into the same sites 2×/week. µCT analysis showed that local administration of curcumin resulted in a complete inhibition of inflammatory bone resorption and in a significant decrease of both osteoclast counts and of the inflammatory infiltrate; as well as a marked attenuation of p38 MAPK and NF-kB activation. We conclude that local administration of curcumin-loaded nanoparticles effectively inhibited inflammation and bone resorption associated with experimental periodontal disease.

## Introduction

Periodontal disease is a chronic inflammatory condition affecting the tissues of support and protection of the teeth and may lead to the destruction of alveolar bone and periodontal ligament. Its prevalence and severity are highly variable in different populations, but it is estimated that 15 to 20% of adult individuals are affected by the more severe forms of the disease and that the less severe forms affect 35 to 60% of the population^1^. Importantly, destructive periodontal disease is a site-specific condition, which frequently affects a limited number of teeth in the dentition. A highly complex microbial biofilm is the necessary extrinsic etiological factor, and currently the pathogenesis is considered a dysbiotic process resulting from an imbalance between microorganisms of the biofilm and the host responses^2^.

Since the immune/inflammatory response is the main culprit for tissue destruction associated with periodontal disease there is a great interest in host-modulatory therapeutic strategies; however in spite of the research efforts and of the immense amount of accrued knowledge on the biological mechanisms of pathogenesis, periodontal treatment is still mostly based on the mechanical removal the calcified and uncalcified bacterial deposits. This approach is somewhat limited as the results are frequently less than satisfactory and highly variable.

Curcumin is the collective denomination for the compound extracted from the rhizomes of the *Curcuma longa* plant, which is widely used as a culinary spice. Chemically, pure curcumin or diferuloylmethane is called (1E,6E)-1,7-bis(4-hydroxy-3-methoxyphenyl) hepta-1,6-diene-3,5-dione^3^, but the raw extract is composed of a mixture of diverse components in variable proportions. Demetoxycurcumin and bis-demethoxycurcumin are the two major biologically active components, which may present distinct and opposing activities^4^. In fact, the diversity of biological effects associated with curcumin described in the vast literature may be related with the variable composition and proportion of the distinct biologically-active components in the rhizome extract collectively denominated ‘curcumin’, which are influenced by the extract/purification methods and the geographical origin of the plant^5,6^.

A multitude of published studies report on anti-inflammatory as well as on wound healing, anti-microbial and anti-neoplastic properties of curcumin, using both *in vitro* and *in vivo* approaches related with diverse conditions such as diabetes, neurological disturbances, cancer, auto-immune conditions and chronic inflammatory conditions including Crohn’s disease, rheumatoid arthritis and periodontal disease^4,7^. A search on the NIH’s public online database of clinical trials (http://www.clinicaltrials.gov) in August, 2017 using the key-word ‘curcumin’ returned 156 registered trials on various clinical conditions such as asthma, multiple myeloma and various other types of cancer, diabetes, Alzheimer’s disease, schizophrenia, inflammatory intestinal diseases. Interestingly, this search returned 9 phase IV clinical trials, including one on the topical use of curcumin as an adjunct in the treatment of periodontal disease. The primary outcome of this single study was the antioxidant activity in the saliva of periodontitis patients, but no results were posted.

There is considerable evidence from both *in vitro* and *in vivo* studies indicating that the anti-inflammatory properties of curcumin attenuate the response of immune cells to periodontal disease-associated bacterial antigens^8–10^ and inhibit periodontal tissue destruction^7,11–13^. However, most of these *in vivo* studies use a systemic route of administration and their results may be limited by the poor pharmacodynamic properties of curcumin, chiefly its hydrophobicity, low absorption rate in the gastrointestinal tract and extremely short plasma half-life^14–16^.

Considering the site-specific characteristic and inflammatory nature of periodontal disease and the anti-inflammatory properties of curcumin, we designed the present proof of principle *in vivo* study to assess the biological effect of the local administration of curcumin in a nanoparticle vehicle in an experimental periodontal disease model.

## Results

### Local administration of nanocurcumin abrogates inflammatory bone resorption in the LPS-induced model of periodontal disease

**Figure 1:**
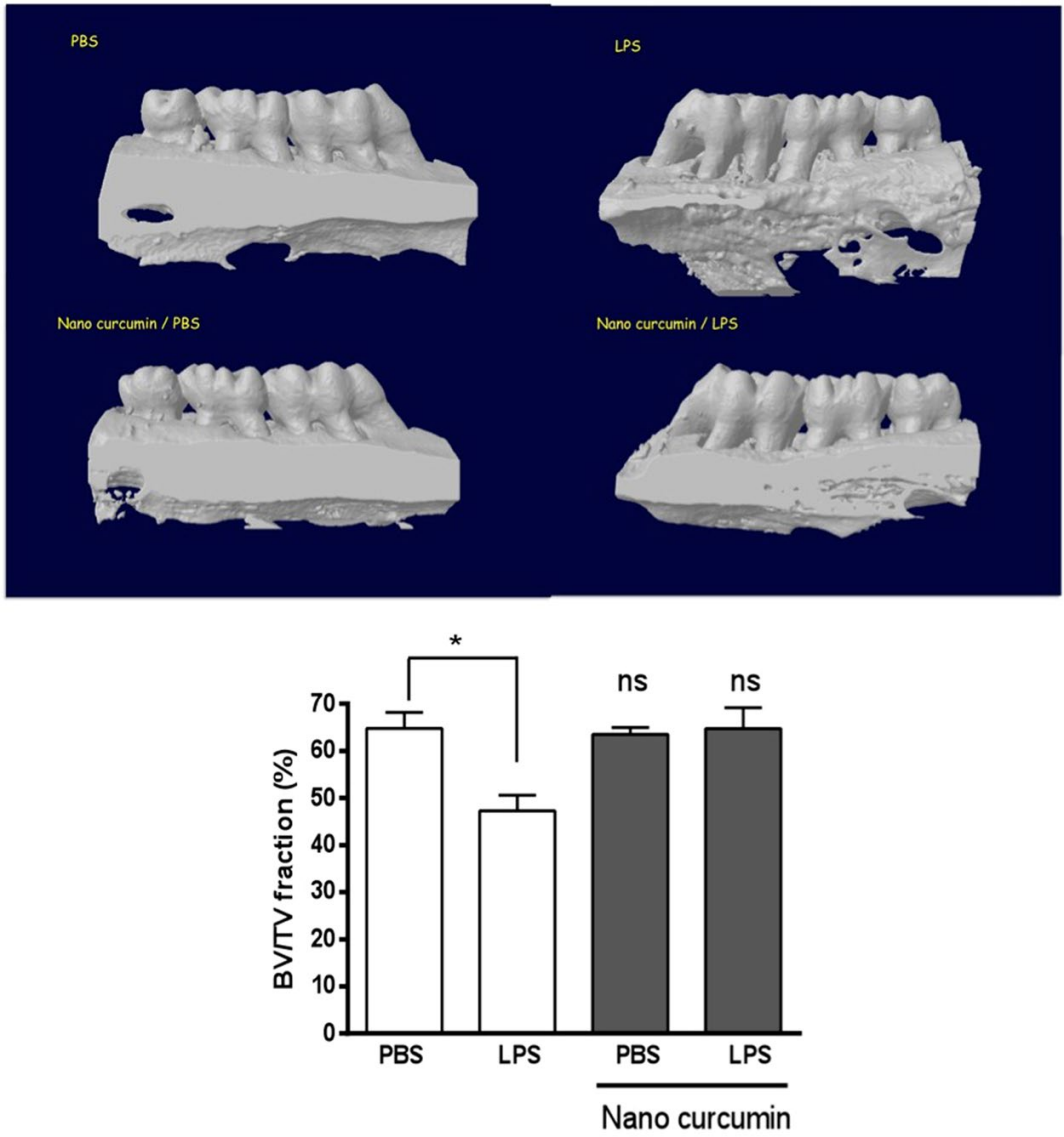
Complete inhibition of bone resorption associated with the LPS model of experimental periodontal disease by local administration of nanocurcumin. Palatal view of representative images of three dimensional reconstructions of the hemi-maxillae in each experimental condition. The bar graph presents the quantitative results of the ratio of mineralized tissue (BV/TV) in the defined standardized ROI, according to the experimental condition. Data from 8 hemi-maxillae of each experimental condition analyzed by ANOVA followed by Tukey post-hoc test (*p < 0.05, ‘ns’ indicates lack of significance in comparison to PBS/vehicle injected hemi-maxillae.

Three dimensional µCT analysis indicates that the BV/TV volume in LPS/vehicle-injected samples was significantly reduced in comparison with the PBS/vehicle-injected samples, demonstrating the effective induction of bone resorption in this experimental model. BV/TV fraction of the samples injected with LPS/nanocurcumin was not statistically different from the PBS/vehicle-injected samples, indicating the absolute inhibition of bone resorption by nanocurcumin administered twice a week in this experimental model. Representative images illustrate the effectiveness of nanocurcumin (Fig. 1).

### Nanocurcumin reduces the number of osteoclasts in the affected area

**Figure 2:**
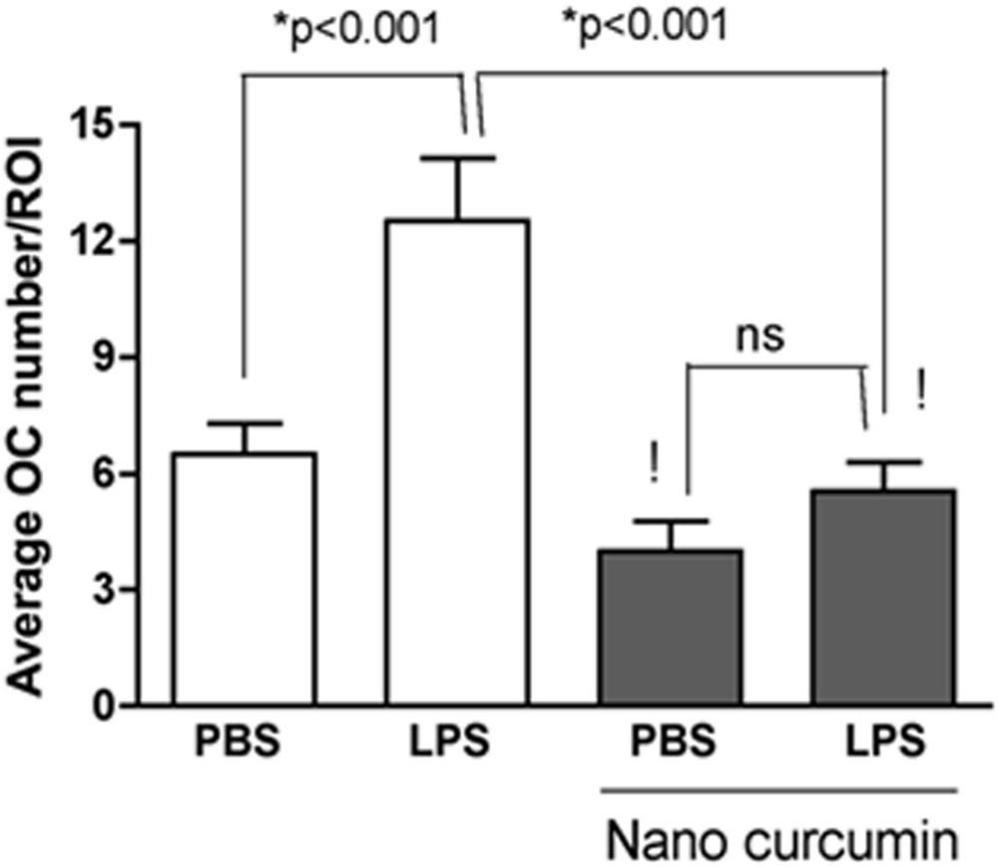
Significant reduction of osteoclast numbers by the local administration of nanocurcumin. Osteoclast counts were significantly increased in the sections of LPS/vehicle-injected hemi-maxillae. Administration of nanocurcumin reduced the numbers of osteoclasts in the defined ROI to the levels observed on sections of hemi-maxillae without disease induction (PBS-injected). Data obtained from two independent counts of a total of 108 sections (27 sections from each experimental condition) and analyzed by ANOVA followed by Tukey post-hoc test (‘ns’: indicates non-significant; ‘!’ indicates lack of significance in the comparison with PBS/vehicle-injected sections).

Since µCT data indicated a complete inhibition of bone resorption in this model, we assessed the number of osteoclasts in a defined area related with the site of injections by morphometric analysis. A significant increase in the numbers of osteoclasts was noted in the LPS/vehicle-injected hemi-maxillae, which corresponds to the decrease on BV/TV and indicates an increase in bone resorption caused by the LPS injections and not affected by administration of the ‘empty’ nanoparticles (vehicle control). The inhibition of bone resorption observed with the administration of nanocurcumin is associated with a significant decrease on the number of osteoclasts in the sections from the hemi-maxillae injected with LPS/nanocurcumin. Moreover, osteoclast counts in the sections of LPS/nanocurcumin-injected hemi-maxillae were not different than those of the sections of PBS-injected hemi-maxillae (Fig. 2).

### Inflammation is attenuated by local administration of nanocurcumin

**Figure 3:**
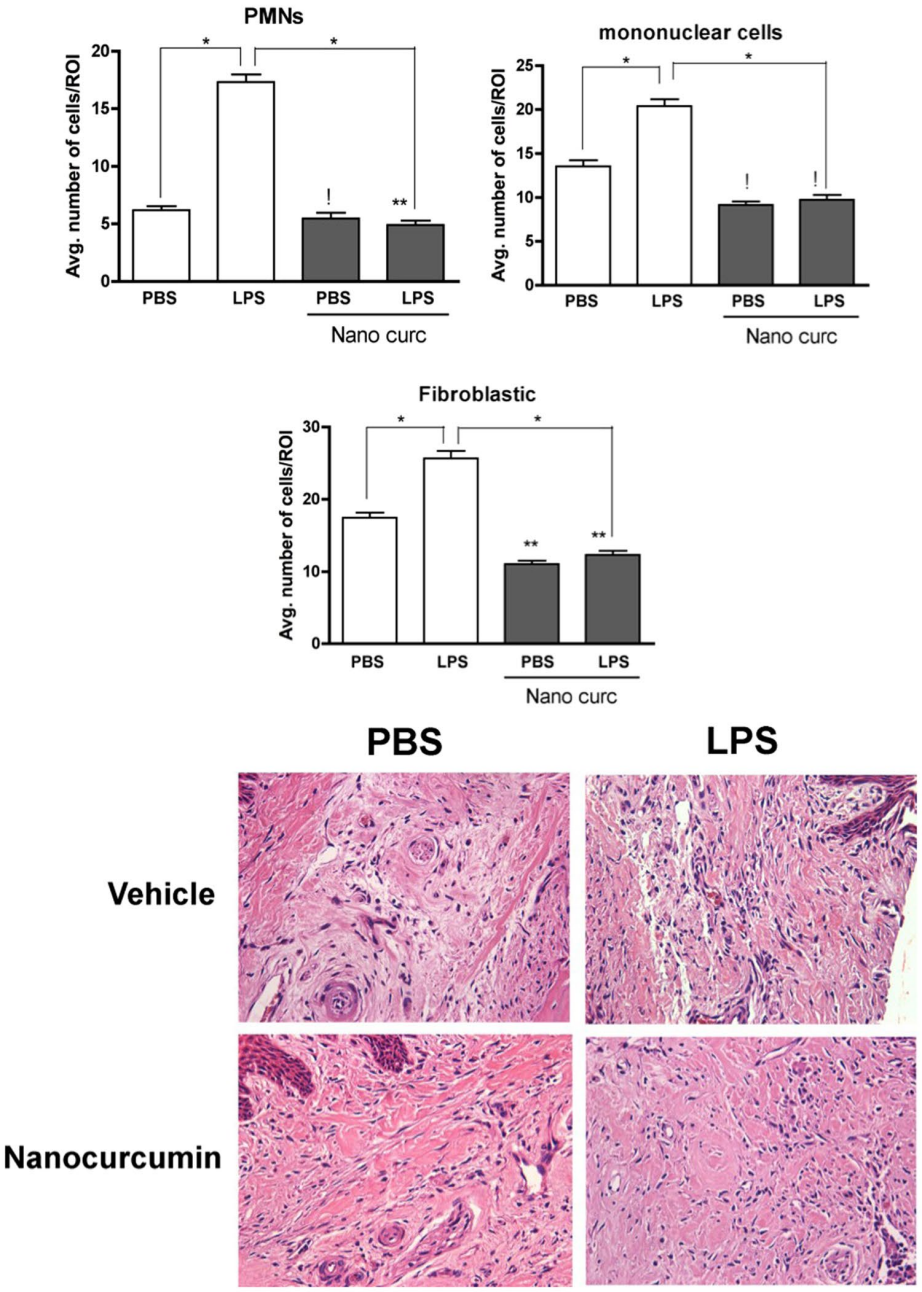
Nanocurcumin reduces the inflammatory cell infiltrate and the numbers of fibroblastic cells. (**A**) Bar graphs present the average number of inflammatory (PMNs, mononuclear cells) and fibroblastic cells counted in 400X images of a defined area of interest of H/E-stained sections according to the experimental condition. Data derived from two independent counts of a total of 180 sections (45 sections/experimental condition) analyzed by ANOVA followed by Tukey post-hoc test. (*p < 0.05 for the comparison between brackted bar, **p < 0.05 in comparison to PBS/vehicle-injected and ‘!’ indicates lack of significance for the comparisons with PBS/vehicle-injected samples). (**B**) Representative images (200X magnification) of the area of interest assessed in H/E-stained sections.

Based on the inhibition of bone loss and on the reduction of osteoclast numbers, we then assessed the severity of inflammation in a defined area corresponding to the site of injections in the gingival tissues since inflammation is the cause of osteoclastogenesis in periodontal disease. Histomorphometric analysis of H/E-stained sections indicated that LPS injections induced a marked inflammatory response, characterized by a significant increase on the numbers of neutrophils (PMNs) and mononuclear cells. Local administration of nanocurcumin caused a significant decrease on the numbers of both PMNs and mononuclear cells, which were comparable to the numbers observed in sections of PBS-injected hemi-maxillae (Fig. 3).

Interestingly, we also observed an increase on the numbers of fibroblastic cells in the sections of LPS/vehicle-injected hemi-maxillae, which was also reduced by the administration of nanocurcumin. In fact, nanocurcumin reduced the levels of fibroblastic cells even in the sections of PBS-injected hemi-maxillae (i.e., without disease induction), supporting this finding as an effect of nanocurcumin (Fig. 3).

**Figure 4:**
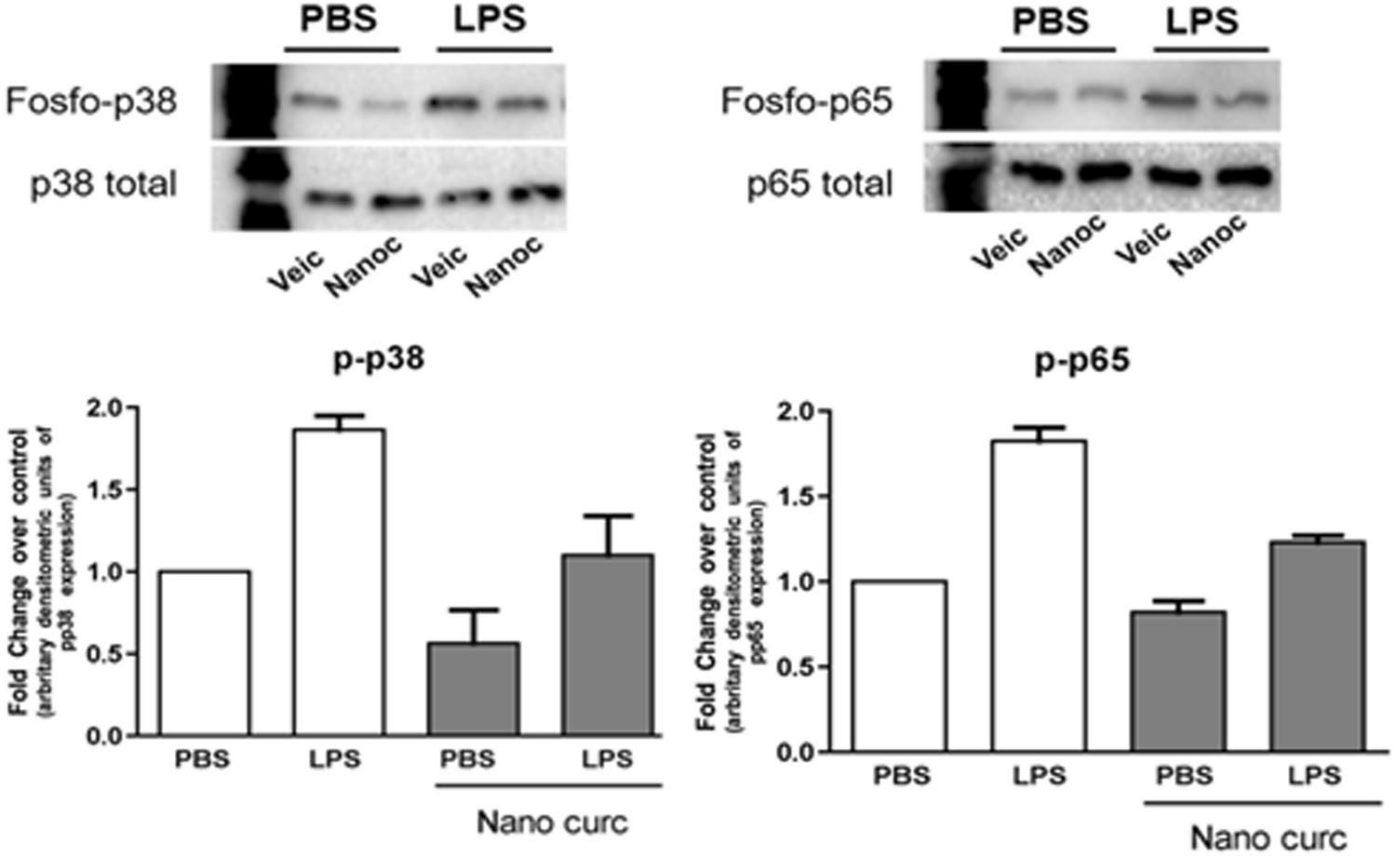
Nanocurcumin attenuates activation of p38 MAPK and NF-kB (p65) in gingival tissues injected with LPS. Representative images of western-blots performed using 40 µg of total protein extracted from the gingival tissues (area of injections) to detect total and activated (phosphorilated) forms of p38 MAPK and p65 (NF-kB). Graphs present data of the densitometric analysis of the bands detected in westerns using protein samples of three hemi-maxillae from each experimental condition. The activation status of p38 MAPK and p65 is shown as relative change (fold change) in comparison with the samples from PBS/vehicle-injected tissues.

The marked reduction on the number of inflammatory cells indicates the anti-inflammatory effect of nanocurcumin. Considering that the experimental model used is associated with a sustained activation of TLR4 by the injections of LPS and that p38 MAPK and NF-kB are major signaling pathways activated downstream of this receptor and also associated with the expression of various pro-inflammatory mediators, we next assessed the effects of nanocurcumin on the activation status of these two signaling pathways in the gingival tissues. Activation of both p38 MAPK and NF-kB was clearly attenuated by the local administration of nanocurcumin, supporting its anti-inflammatory effects (Fig. 4).

## Discussion

This is an initial, ‘proof of concept’ type of study to assess the viability and biological effect of local administration of curcumin in a nanoparticle formulation. The results indicate the effective inhibition of inflammatory bone resorption, which was associated with a significant reduction in both osteoclast numbers and on the number of infiltrating inflammatory cells. Activation of p38 MAPK and NF-kB in the gingival tissues was also markedly attenuated. Importantly, we have not observed any adverse effects, either local or systemic, associated with the administration of the nanocurcumin or of the nanoparticle ‘empty’ vehicle.

Curcumin’s anti-inflammatory properties are widely documented by many *in vitro* studies assessing variety of cell types, including macrophages^17,18^, dendritic cells^19,20^, neutrophils^21,22^, and T^23,24^ and B cells^25,26^. Numerous studies published in diverse *in vivo* models, such as adverse pregnancy outcomes due to placental inflammation^27^, lung inflammation^28^, asthma^29^, sepsis^30^, intestinal inflammation^31^, osteoarthritis^32^ and psoriasis^33^ also document the anti-inflammatory properties of curcumin.

Specifically in the context of periodontal disease, our research group reported that systemic administration of curcumin in lipid vehicle inhibited activation of NF-kB (but not of p38 MAPK) and inflammatory bone resorption in the same LPS-induced experimental model of periodontal disease^7,11^. Similar results are observed with the systemic administration of curcumin in the ligature model of experimental periodontal disease^12,13^. Recently, the inhibition of bone resorption by systemic administration of curcumin in the ligature model was associated with decreases on the expression of IL-17 and RORγT^34^.

Most of the *in vitro* studies use curcumin in non-acqueous vehicles (e.g., ethanol or DMSO), whereas *in vivo* studies uses systemic administration of curcumin in lipid or in a polar compound (e.g., carboxymethylcellulose) due to the virtual insolubility of curcumin in acqueous vehicles. This fact is one of the curcumin’s chemical characteristics which limits its clinical use. The poor pharmacodynamic properties, including the reduced absorption rate in the gastrointestinal tract and the plasma half-life in the order of a few minutes^16^ have driven the search for chemical modifications in the compound that improve its pharmacodynamic properties and even enhance the biological activity^35^,^36^. Another strategy intensely studied to improve the pharmacodynamic and biological properties of curcumin is the use of alternative vehicles, particularly lipid-, chitosan- or hydrolized corn protein-associated nanoparticle formulations^37–39^. A proprietary nanoformulation of curcumin (‘Curcumin Rich Theracurmin’, Natural Factors Inc.) increased the half-life or curcumin in the plasma of humans from minutes to more than 9 h after a single administration^40^.

We used nanoparticles synthesized from polylatic acid and co-glycolic acid, which has been shown to increase 15 times the half-life of curcumin in the plasma of rats^41^. These nanoparticles also allow for chemical modifications that may direct its absorption by a given tissue or cell type, alter the absorption process to avoid macropinocytosis and possible lipossome degradation or allow the tracking of its sub-cellular localization by covalently binding with fluorescent molecules^42^.

Fewer studies have assessed the local administration of curcumin in periodontal disease, and interestingly all of these are clinical studies. The local application of curcumin in slow-releasing vehicles (80% of the curcumin content released in 72 h) resulted in positive anti-microbial and anti-inflammatory effects, indicated by a significative decrease on the gingival index^43^. Curcumin used as a mouthwash associated with mechanical periodontal therapy in chronic gingivitis patients resulted in improvements on plaque and gingival indexes that were similar to those obtained with a chlorhexidine mouthwash^44^. The local application of collagen strips containing 0.2% curcumin as an adjunct to mechanical periodontal therapy significantly reduced the activity of superoxide dismutase (SOD) in the GCF; however all clinical parameters were similar to those associated with mechanical therapy alone^45^. Nevertheless, most of these studies have design issues (e.g., absence of a vehicle or placebo control, lack of blinding of the examiner to the experimental treatment) that can introduce biases in the results and reduce their external validity. Moreover, not all studies clearly indicate the source of the natural curcumin used, which coupled with the pharmacodynamic issues of this compound and with variations in the concentrations and vehicle used make the evidence inconsistent and anecdotal.

Our data demonstrate the biological effect of locally administered nanoparticle formulation of curcumin in the LPS-induced model of experimental periodontitis; however one must consider the limitations of the experimental approach. Injections of LPS from *E. coli* in the gingival tissues has demonstrated to induce the alveolar bone resorption *in vivo*, increase the levels of proinflammatory mediators and collagen degradation in gingival tissues^11,46–49^, which are the main features of periodontal disease in humans. LPS from *E. coli* is an important activator of TLR4 (Toll-like receptor 4), which is involved in the innate response to stimulation by bacterial products in the periodontal pocket^50^ and it has a critical role for LPS-induced bone resorption^51,52^. In addition, LPS model provides control over the intensity of the inflammatory stimulus, which is maintained throughout the experimental period, allowing for the development of chronic inflammation, involving both innate and adaptive immunity. However, there are limitations in this model and experimental approach, as both the inflammatory stimulus (LPS) and the treatment (nanocurcumin) are injected directly into the gingival connective tissue, bypassing the involvement of sulcular and junctional epithelium. Also, there are no other bacterial-derived antigens, such as toxins or other microbial-associated molecular patterns that could activate other pattern-recognition receptors (PRRs). We assessed the effects of nanocurcumin on inflammation by histomorphometric analysis, which is not as specific as an immunohistochemical approach and also subjected to bias introduced by variations in the angle of sectioning that may alter the perception of nuclear morphology. Albeit limited, this approach has been used in many studies^53–60^, moreover, if we consider the total cellular infiltrate (without the distinction of different cell types based on the nuclear morphology), the local administration of curcumin reduced the number of cells in the gingival tissue, which is consistent with a general inhibition of inflammation. This observation is supported by the clear decrease of p38 MAPK and NF-kB activation in the gingival tissues assessed by western blot. This analysis is also limited by the reduced ‘n’, which prevented a proper statistical analysis. Also, since cytosolic proteins represent a minor fraction of total proteins isolated from fibrous connective tissues (such as the gingival tissues), slight variations in the area from which the tissues were harvested (i.e., the inclusion of less inflamed areas) may have also affected the results.

We have chosen p38 MAPK and p65 (NF-kB) as ‘general indicators of inflammatory activity’ because these are the two major signaling pathways activated downstream of TLR4 (activated by the LPS used as inflammatory stimulus in our model) and also involved in the expression of multiple inflammatory genes^61–63^. Also, our research group has previously shown the activation of these two signaling pathways in this experimental model^46^. There is evidence indicating that curcumin inhibits TLR4 signaling^64–66^ and even the dimerization of this receptor^61^. However, we cannot rule out the possibility that the biological effects of locally administered curcumin may be associated with the modulation of other signaling pathways and biological mechanisms. In fact, curcumin has been shown to reduce the oxidative stress and the activity of other signaling pathways including JAK/STAT, SOCS1 and 3, ERK and JNK MAPKinases, PI3K/Akt^8,31^, as well as the activity of inflammassomes^67^ and the expression of microRNAs^68^.

The decrease of fibroblastoid cells observed is somewhat intriguing, particularly because it was observed even in the absence of experimentally-induced inflammation. This may represent a potential negative effect on tissue repair or turnover, but there is abundant evidence indicating a favourable effect of topically applied curcumin in wound healing^69,70^. We speculate that the decrease in fibroblastic cells may be related with the anti-inflammatory effect of nanocurcumin, since increased fibroblast proliferation is associated with inflammation^71^ and even the control specimens in our model may present some basal level of inflammation associated with the indigenous bacterial biofilm and/or with the mechanical trauma from the microinjections. In fact, it is possible that the overall reduction in the activity of p38 MAPK and NF-kB observed in the gingival tissues is related with a change in the phenotype of the fibroblasts in the gingival tissues, since these cells express inflammatory cytokines and metalloproteinases in inflamed microenvironments^72^.

In conclusion, this proof of principle study demonstrates that local application of curcumin in nanoparticle vehicle in the LPS-induced model of experimental periodontal disease inhibits inflammatory bone resorption, which is associated with a reduction in osteoclast numbers and inflammation. It is important to note that the perspective of clinical application is based on the non-invasive, topical application of the nanocurcumin in the gingival sulcus of diseased areas as an adjunct of mechanical periodontal treatment. This perspective of clinical application requires further studies regarding the local absorption of the nanoparticled curcumin and may also involve further developments that may improve its efficacy, such as photoactivation to enhance its anti-microbial properties.

## Methods

### Curcumin nanoparticles

Nanoparticles were prepared by Dr. Antonio Claudio Tedesco (Department of Chemistry, University of Sao Paulo at Ribeirão Preto). Curcumin was obtained commercially (Sigma-Aldrich Co. cat# C1386, Lot# 081M1611V). A 1:1 ratio of poly-lactic and polyglycolic acids (obtained from Sigma-Aldrich Co.) were loaded with 0.05 mg/mL of curcumin (ethanol solution). The vehicle control (‘empty’ nanoparticles) were prepared using the same method, but using loaded only with the same volume of ethanol vehicle. Briefly, the polymers were dissolved in di-chloromethane and added to a 1% acqueous solution containing polyvinyl alcohol and curcumin. The mixture was submitted to vigorous agitation for emulsification of water in oil. The organic solvent was removed by agitation at room temperature followed by evaporation under reduced pressure. The particles were centrifuged (1,100–4,600 *g*, 4 C) and, after three washes with distilled water, resuspended in PBS and filter-sterilized. Nanocurcumin and the empty nanoparticle vehicle were stored protected from light at 4 C and used within 30 days of its preparation.

### Animals and *in vivo* experiment

A total of 16 Holtzman rats (*Rattus norvegicus albinus, Holtzman*) were used. Animals were housed in microisolation cages in groups of 4 and kept in standardized conditions of temperature and humidity (21±1 C, 65–70%) and 12 h light/dark cycle in the animal facility of the School of Dentistry at Araraquara-Univ Estadual Paulista (UNESP), according to the recommendations of the brazilian National Council for the Control of Animal Experimentation (CONCEA). The experimental protocol was approved by the Institution’s Ethics Committe on the Use of Animals (CEUA). Water and standard commercial laboratory rat feed were provided *ad libitum*.

Experimental periodontal disease was induced by local microinjections of 3 µL of a 10 mg/mL LPS solution (from *Eschericia coli*, strain 055:B5 - Sigma Chem Co.) into the gingival tissues adjacent to the palatal surface of both upper first molars, as described by us previously^11,46–48^. Control animals were injected with the same volume of PBS (pH 7.4, no Ca/Mg, used to dilute the LPS) in the same anatomical regions. These injections were performed 3 times per week, for 4 weeks by a trained operator using Hamilton-type microsyringes and customized needles of 30 Ga and 0.6 cm length (Hamilton Robotics - Agilent Technologies). The 16 rats were divided into two groups of 8 animals, according to the bilateral injection of LPS or PBS.

**Figure 5:**
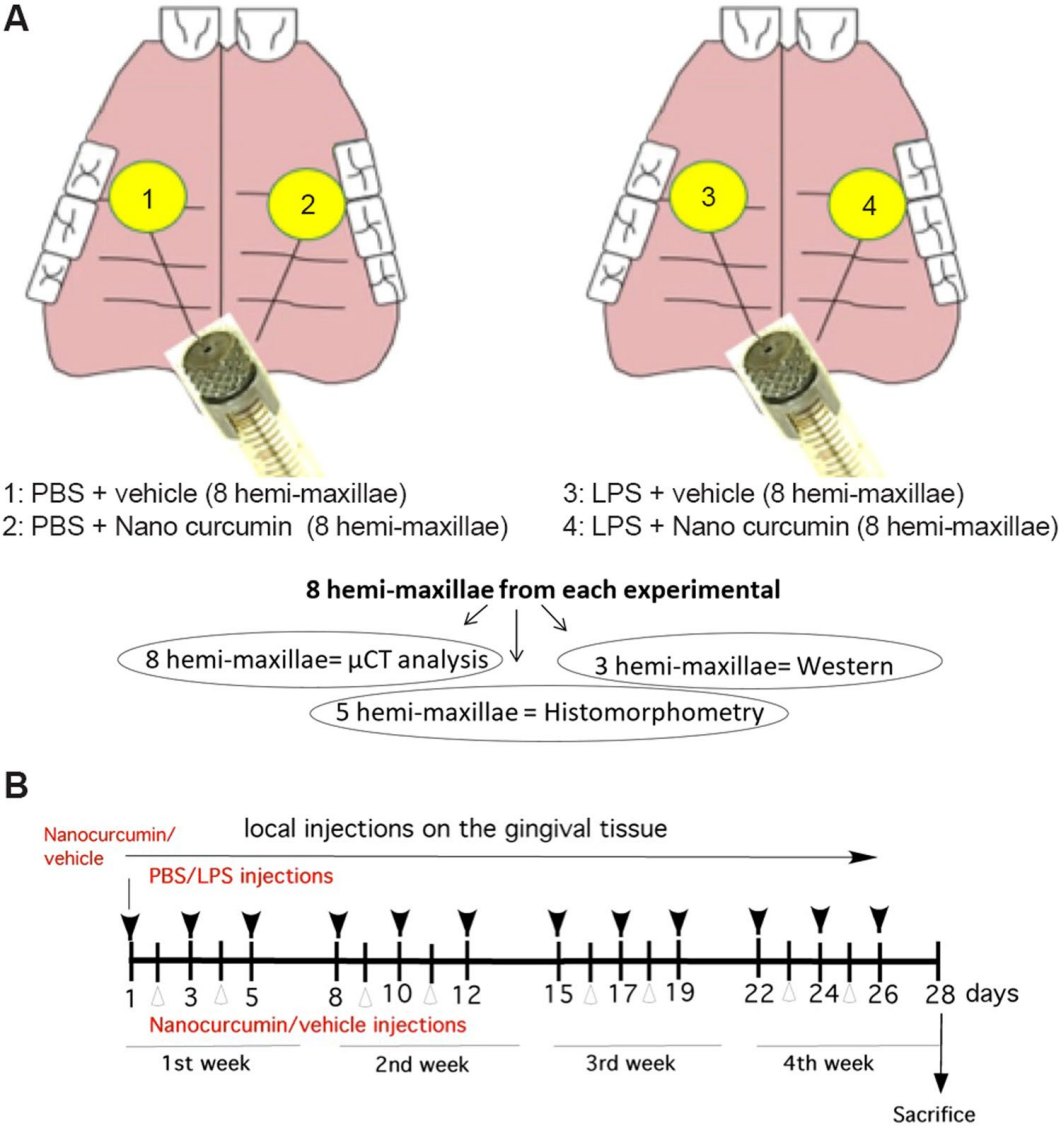
Experimental approach for the induction of experimental periodontal disease and local treatment with nanocurcumin. (**A**) Schematic representation of the experimental approach and distribution of the samples according to the various analyzes: bilateral injections of either LPS or PBS (vehicle control) were performed 3 times a week. Twice a week, contra-lateral injections of either nanocurcumin (left side of the animal) or vehicle (right side of the animal) were performed. Considering the use of 16 rats, this approach generates 8 samples for each experimental condition. All samples were submitted to µCT analysis and then, 3 hemi-maxilla from each experimental condition were destined to western blot, and the other 5 hemi-maxillae were used to histomorphometric analysis. (**B**) Schematic representation of the ‘time line’ for the 28-day period of the *in vivo* experiment: black arrows indicate the days in which either LPS or PBS were injected (3×/week) and open arrows indicate the days in which either nanocurcumin or vehicle were injected (2×/week).

Three microliters of nanocurcumin or of the ‘empty’ nanoparticles (vehicle control) were injected contra-laterally (nanocurcumin on the left side and nanovehicle on the right side of the animal) into the gingival tissues at the same anatomical areas twice a week, on the days following the first and second injections of either LPS or PBS (Fig. 5).

All animals were sacrificed at the end of the 28-day experimental period by cervical dislocation under dissociative anesthesia. The hemi-maxillae were carefully ressected. The soft tissue from the area in which the injections were performed was obtained from 12 hemi-maxillae (3 from each experimental condition), immediately frozen in liquid nitrogen and kept at −80C until used. The remaining 20 hemi-maxillae (5 from each experimental condition) were fixed in 4% paraformaldehyde for 24 h at 4 C and transferred to 70% ethanol for µCT scanning alongside with the 12 hemi-maxillae from which soft tissue was ressected. After scanning on the µCT, the 20 hemi-maxillae that still had the intact soft tissue were submitted to routine processing (including decalcification in 0.5% EDTA, pH 8.0) for paraffin embedding, sectioning and H&E staining.

### µCT analysis

Scanning was performed in 18 µm slices, using pre-optimized settings (50 kV, 300 mA, 0.5 mm aluminum attenuation filter) on a Skyscan microtomograph (Skyscan, Aartselaar, Belgium). After tridimensional reconstruction, the resulting images were rotated in a standardized tridimensional orientation using defined anatomical references. A standardized threshold was set for the distinction or non-mineralized and mineralized tissues and a defined region of interest (ROI) of 1,400 × 500 × 540 µm (length × height × thickness) was set and positioned on the images according to anatomical landmarks. The fraction of mineralized tissue in the total volume of this ROI (BV/TV) was calculated using CT Analyser software (version 1.12.4.0 Bruker microCT, Skyscan, Belgium).

### Histomorphometric analysis

The histomorphometric analysis was performed to assess the effect nanocurcumin on the inflammatory process in the gingival tissues. Initially, a 500 µm line starting from the bottom of the junctional epithelium was drawn towards the center of the palate on a digital image obtained at 50× magnification. At the end of this 500 µm line, a defined landmark was used to position the field of image at 400× magnification. The whole microscopic field at 400× was used for the assessment of the inflammatory process. A total of 9 semi-serial sections (5 µm thick) obtained in the frontal (buccal-palatal) plane were assessed for each hemi-maxilla, including three histological sections from each of three different regions spaced by 250 µm in the sagital/mesio-distal plane: ‘anterior region’, corresponding to the mid-portion of the upper first molar; ‘central region’, corresponding to the proximal area between first and second upper molars; and a ‘posterior region’, corresponding to the mid-portion of the second upper molar. Five hemi-maxillae from each experimental condition were used in this analysis, resulting in a total of 45 sections for each experimental condition (180 sections in total). Polymorphonuclear neutrophils, mononuclear cells and fibroblastic cells were differentially counted based on the nuclear morphology on the 400× microscopic field by a trained and experienced examiner, who was blind to the experimental condition.

Osteoclasts, defined as large cells near the surface of the bone containing three or more nuclei, were counted on the surface of alveolar bone surface beginning at the end of the 500 µm line and extending up to the apex of the palatal root (Fig. 2). For osteoclast counting, 9 sections (3 sections from each of the three regions: ‘anterior’, ‘central’ and ‘posterior’) were analyzed for each hemi-maxilla. Three hemi-maxillae (27 sections) were analyzed for each experimental condition, resulting in a total of 108 sections assessed. The same trained and experienced examiner counted the osteoclasts, also without knowledge of the experimental condition. Both analyses were performed twice, with an interval of 6 weeks between assessments.

### Western Blot

Total protein was extracted from the frozen gingival tissue sections ressected from 3 hemi-maxillae from each experimental condition. Tissue samples were mechanically disrupted with a plastic pestle in 150 µL of lysis buffer (M-Per, Pierce - ThermoFisher Scientific) supplemented with protease and phosphatase inhibitor cocktails (Complete and PhosStop, Roche Life Science). Samples were centrifuged (12,000 RPM, 4 C, 10 min) and the supernatant was transferred to new microfuge tubes and quantitated using the Bradford method (Bio-Rad Laboratories). Forty µg of total protein from each sample were mixed with sample buffer containing 100 mM DTT, further denatured by heat (95 C, 5 min), loaded onto discontinous 4–12% polyacrilamide gels and submitted to SDS-PAGE electrophoresis (100 V constant current, 60 min) and blotting onto 0.2 µm nitrocellulose membranes (110 mA/gel, 60 min). Primary antibodies for total and phosphorilated forms of p38 MAPK and p65 (Cell Signaling Inc.) were incubated at the recommended dilution for 18 h at 4 C. Following incubation with secondary HRP-conjugated secondary antibodies, a chemiluminescent substrate (SuperSignal West Pico, Pierce - ThermoFisher Scientific) was used and the membranes were visualized using a digital documentation system (GelDoc XT, Bio-Rad Laboratories). The images of the membranes were submitted to densitometric analysis using the ImageQuant (Bio-Rad Laboratories), using the density of the bands corresponding to the total (phosphorilated and non-phosphorilated) forms of the proteins of interest used for normalization of the active (phosphorilated) forms.

### Statistical analysis

Data obtained from each experiment was analyzed using GraphPad Prism 5.0 (GraphPad Software Inc., San Diego, CA, USA). The objective of the analysis was to compare the results according to the different experimental conditions. As indicated in the figure legends, we used unpaired Student t tests with Welch’s correction for unequal variances and ANOVA with post-hoc Tukey test for pairwise comparisons. Significance level was set at 95% (p < 0.05) in all analysis.

### Data availability

All data generated or analyzed during this study are included in this published article.
